# Effect of Processing on the In Vitro and In Vivo Protein Quality of Beans (*Phaseolus vulgaris* and *Vicia Faba*)

**DOI:** 10.3390/nu10060671

**Published:** 2018-05-25

**Authors:** Matthew G. Nosworthy, Gerardo Medina, Adam J. Franczyk, Jason Neufeld, Paulyn Appah, Alphonsus Utioh, Peter Frohlich, James D. House

**Affiliations:** 1 Department of Food and Human Nutritional Sciences, University of Manitoba, Winnipeg, MB R3T 2N2, Canada; matthew.nosworthy@umanitoba.ca (M.G.N.); medinag@myumanitoba.ca (G.M.); umfranc3@myumanitoba.ca (A.J.F.); jason.neufeld@umanitoba.ca (J.N.); 2 Food Development Centre, Portage la Prairie, MB R1N 3J9, Canada; Paulyn.Appah@gov.mb.ca (P.A.); Alphonsus.Utioh@gov.mb.ca (A.U.); 3 Canadian International Grains Institute, Winnipeg, MB R3C 3G7, Canada; pfrohlich@cigi.ca; 4 Richardson Centre for Functional Foods and Nutraceuticals, University of Manitoba, Winnipeg, MB R3T 6C5, Canada; 5 Canadian Centre for Agri-Food Research in Health and Medicine, University of Manitoba, Winnipeg, MB R2H 2A6, Canada; 6 Department of Animal Science, University of Manitoba, Winnipeg, MB R3T 2N2, Canada

**Keywords:** baking, cooking, extrusion, in vitro protein digestibility, beans

## Abstract

In this work, the protein quality of different bean types after undergoing the preparatory methods of baking, cooking and extrusion was assayed. Protein quality was assessed using a rodent bioassay to evaluate growth and protein digestibility while amino acid composition was determined via HPLC. In vivo protein digestibility was compared to an in vitro assessment method. The average protein digestibility corrected amino acid score (PDCAAS) for processed beans was higher than the digestible indispensable amino acid score (DIAAS) (61% vs. 45%). Extrusion/cooking of Phaseolus varieties resulted in higher PDCAAS (66% on average) and DIAAS values (61% on average) than baked (52% and 48%) while baked faba beans had higher PDCAAS (66%) and DIAAS (61%) values. A significant correlation was found between PDCAAS and in vitro PDCAAS (*R*^2^ = 0.7497). This demonstrates which bean processing method will generate the optimal protein quality, which has benefits for both industrial production and individual domestic preparation.

## 1. Introduction

The common dry bean, *Phaseolus vulgaris*, is comprised of many different varieties and is the largest pulse crop with an annual production of over 26 million tons worldwide [1]. Of these different varieties of beans, including navy, red kidney, pinto and black, the pinto bean is the most commonly consumed variety in the United States [2]. Beans are known to be high in protein, contain iron, calcium, folic acid, and other vitamins in addition to having low fat content [3]. The protein content of beans can vary from 21.4 to 23.6%, and the first limiting amino acid can differ depending upon bean variety [4]. Previously it has been found that tryptophan is limiting in navy beans, black beans, and pinto beans while methionine is limiting in red kidney beans [5,6]. Faba beans (*Vicia faba*), although a bean in a broad sense of the term, are different than beans from *Phaseolus vulgaris* mainly in protein content. The protein content of faba beans is typically between 27–34% [7,8], with the limiting amino acid being either tryptophan or the sulfur amino acids, depending on variety [5,7,8]. As all pulses, including beans, are known to contain anti-nutritive factors such as trypsin inhibitors, tannins and hemagglutinins, raw beans must undergo processing prior to human consumption [9,10]. Processing methods such as extrusion, boiling or baking, does not only alter and inactivate anti-nutritive factors, but may also alter the digestibility or amino acid content of bean flour.

Various forms of extrusion are commonly used in the production of snacks and pasta. It is a process where flour is moved through a machine via a screw system under a specific set of moisture, temperature and pressure conditions. This process has been shown to reduce the activity of trypsin/chymotrypsin inhibitors, as well as reducing hemaglutinnin content [11]. The digestibility of bean protein does increase after extrusion, potentially due to the reduction of anti-nutritive factor activity [12,13,14]. Extrusion does not alter the protein content of beans, however, the amino acid composition can be affected [12,13,15,16].

Cooking, or boiling, of beans is perhaps the most common method of preparing beans in a home setting. Both trypsin inhibitor activity and the concentration of tannins, another anti-nutritive factor, are decreased after cooking leading to an increase in protein digestibility [17]. Cooking can increase protein content in kidney beans, chickpeas, and faba beans potentially through the loss of carbohydrates during the cooking process [6,18,19,20,21]. With respect to amino acid composition, some studies have found increased concentrations of essential amino acids after cooking, while others have found reduced concentrations of methionine, tyrosine and threonine [19,22,23]. Baking is another form of heat processing that is used for cooking beans and legumes, although in a laboratory setting autoclaving is occasionally used as a surrogate for a standard baking procedure. A study using faba beans found that there was a reduction in both hemagglutinin and trypsin inhibitor activity after autoclaving [24]. Although these methods of preparation have shown reduction in anti-nutritive factors, direct investigation as to the impact of these processes on the protein quality of multiple bean varietals has yet to be performed.

Three different measures of protein quality were quantified: the Protein Digestibility Corrected Amino Acid Score (PDCAAS), the Digestible Indispensable Amino Acid Score (DIAAS) and the Protein Efficiency Ratio (PER) [25,26,27]. At present, the United States uses PDCAAS for the regulation of protein content claims while Canada requires the determination of PER. The most recent protein quality measurement recommended, DIAAS, has yet to be incorporated into any regulatory system. In conjunction with these in vivo measurements of protein quality, an in vitro method for determining protein digestibility was also performed to determine an in vitro PDCAAS value [28]. The aim of this study was to determine what effect extrusion, boiling and baking have on the protein quality of different types of beans (black, faba, navy, pinto and red kidney).

## 2. Materials and Methods

The University of Manitoba’s Institutional Animal Care Committee approved all procedures, as mandated by the Canadian Council on Animal Care [29].

### 2.1. Chemicals

All chemicals and reagents were purchased from Sigma (Oakville, ON, Canada).

### 2.2. Sample Procurement and Preparation of Extruded, Baked and Cooked Flours

Samples of beans were provided by SaskCan Pulse Trading (Regina, SK, Canada), Thompsons Ltd. (Blenheim, ON, Canada), Diefenbaker Seed Processors (Elbow, SK, Canada), Hensall District Cooperative (Hensall, ON, Canada), Viterra (Bow Island, AB, Canada) and Legumex Walker (Winkler, MB, Canada). Samples of faba beans were provided by SaskCan Pulse Trading (Regina, SK, Canada). After procurement, and prior to processing, samples of similar beans from different suppliers were combined and thoroughly mixed. Milling of uncooked beans, extrusion of the resulting flour and milling of the extrudate was performed as previously described [30]. Flours were extruded at 45–55 kg/h and 0.7–1.2 kg/h, dry and liquid feed rates respectively, through a cracker shaped die. The screw speed was 400–600 rpm. The extrusion barrel temperatures were: 30–50 °C, 70–90 °C and 100–120 °C. The cooking process was performed as previously described [30], The baking process was also performed as previously described, however an additional processing step was incorporated: 1.24–1.79 kg of extruded pieces (≈12.5 mm diameter) were transferred to 7–9 sheets and baked for 27–35 min after resting [30].

### 2.3. Analytical Procedures

Percent crude protein (CP; N× 6.25) was determined via Dumas Nitrogen Analyzer (Dumatherm DT, Gerhardt Analytical Systems, Königswinter, Germany), percent dry matter (DM) and ash were determined [31]. The percent crude fat was determined by hexane extraction and gravimetrics. Methionine and cysteine were determined using the AOAC Official Method 45.4.05, while other amino acids, except tryptophan, were determined according to AOAC Official Method 982.30

### 2.4. Tryptophan Analysis

Tryptophan content was determined using alkaline hydrolysis [32]. In brief, sample (40 mg), double distilled water (14 mL) and barium hydroxide octahydrate (8.4 g) were added into a polypropylene flask which was capped loosely and autoclaved at approximately 110 °C for 20 h. After being removed from the autoclave, 30 mL deionized water, 1.0 mL of 0.054% alpha-methyl tryptophan, and 5.0 mL of 0.5 M orthophosphoric acid were added and the final solution was brought to a pH of 3.0 with 6 N HCl. Finally, 20 mL of methanol was added, and the solution brought to a final volume of 100 mL with deionized water. Samples were analyzed at room temperature on a Varian HPLC using a 125 mm × 4 mm C18 Luna column. The mobile phase consisted of 0.3% acetic acid, 0.05% 1,1,1-trichloro-2-methyl-2-propanol and was brought to a pH of 5.0 using ethanolamine. The flow rate was 1 mL/min with an excitation wavelength of 280 nm and emission wavelength of 356 nm.

### 2.5. Protein Digestibility-Corrected Amino Acid Score

Diets were formulated to contain 10% protein, 10% total fat, and 5% cellulose with the remaining energy derived from corn starch. Vitamins (AIN-93 formulations; Harlan Teklad, Madison, WI, USA) and minerals (AIN-93G Mineral Mix, Dyets, Bethlehem, PA, USA) were added to diets to meet the micronutrient requirements of laboratory rats. Male weanling Sprague–Dawley laboratory rats (*n* = 160, 10 per treatment; initial weight 70 g) were procured from Charles River (Charles River Laboratories, Wilmington, MA, USA), randomly assigned to one of 16 diets, either casein or one of the baked/cooked/extruded treatments of five bean types, individually housed in suspended wire-bottomed cages with absorbent paper underneath to retain the feces for collection as well as any spilled diet, and treated as previously described [30]. Water was provided ad libitum, with feed being restricted to 15 g/day for a 4-day acclimation period as well as a 5-day balance period during which feed intake was recorded. During the balance period feces was collected, dried and assayed for dry matter and nitrogen content.

True protein digestibility (TPD%) was calculated using the following equation:

TPD% = ((Nitrogen Intake − (Fecal Nitrogen Loss − Metabolic Nitrogen Loss))/Nitrogen Intake) × 100(1)

where nitrogen intake is determined via diet consumption, fecal nitrogen loss by fecal nitrogen, and metabolic nitrogen loss by determining fecal nitrogen in rats consuming a protein free diet.

The PDCAAS was calculated as:

PDCAAS (%) = TPD% × Amino Acid Score (AAS)(2)

where the AAS is determined by comparing the amino acid composition of the protein source to the reference pattern recommended by the FAO/WHO, the amino acid requirements of a 2–5 year-old child [25]. The lowest amino acid ratio is considered as the AAS.

### 2.6. Digestible Indispensable Amino Acid Score (Diaas)

DIAAS was calculated using the amino acid reference pattern for children aged 6 months to 3 years, as recommended by the FAO/WHO [26]. The same TPD% as that determined for PDCAAS was used to determine individual amino acid digestibilities. While it is preferable that the ileal amino acid digestibility be used, the use of fecal digestibility is considered acceptable (FAO/WHO, 2013). The calculation of DIAAS is described below [26]:

DIAAS % = 100 × ((mg of Digestible Dietary Indispensable Amino Acid in 1 g of the Dietary Protein)/(mg of the Same Dietary Indispensable Amino Acid in 1 g of the Reference Protein))(3)

### 2.7. In Vitro Protein Digestibility-Corrected Amino Acid Score (Ivpdcaas)

An in vitro digestibility assay was also performed on each protein containing ingredient [28,33,34]. Briefly, the equivalent of 62.5 mg of protein was heated to 37 °C and adjusted to a pH of 8.0. The samples were then monitored for 10 min to record the stability of the pH, followed by the addition of a multi-enzyme cocktail containing trypsin, chymotrypsin and protease. After the addition of the digestive cocktail, the subsequent pH drop was recorded for 10 min. The in vitro protein digestibility was calculated as follows, where the ΔpH10 min is the change in pH in 10 min from the initial pH of about 8.0.

In Vitro Protein Digestibility (IVDP)% = 65.66 + 18.10 ∙ ∆pH (10 min)(4)

The IVPDCAAS was calculated as a product of the AAS, as determined for PDCAAS, and IVPD%.

### 2.8. Protein Efficiency Ratio (Per)

A four-week feeding trial is recommended by Health Canada to determine PER [27]. The same animals used for the determination of PDCAAS were also used for the determination of PER. For the current study, diet intake and bodyweights were recorded over a period of four weeks, including the 9-day protein digestibility study period used to determine PDCAAS. The Protein Efficiency Ratio was calculated as follows:

PER = Amount of Weight Gain (g)/Amount (g) of Protein Consumed(5)

Values were also adjusted to a standardized 2.5 PER value for the reference casein.

### 2.9. Statistics

Results were compared via two-way ANOVA with post-hoc analysis using Tukey’s multiple comparison test, while the relationship between in vivo and in vitro digestibilities and corrected amino acid scores was determined via regression analysis (GraphPad Prism, 7.0, GraphPad Software, La Jolla, CA, USA).

## 3. Results and Discussion

### 3.1. Proximate Analysis

Dry matter, fat and protein content for the processed bean samples are presented in Table 1, with fat and protein presented on a dry matter basis. The dry matters of the untreated flours were similar across all bean types and similar to previously reported results [35]. The dry matter of the processed flours is similar across all bean types, with a minimum of 95.13%, found in both baked and cooked pinto, and maximum of 100% for cooked black bean. Extruded flours had the lowest fat percentage in all bean types, ranging from 0.24% for black bean to 1.50% for navy bean. Cooked black, navy and pinto bean had the highest fat percentage (1.61%, 1.71% and 1.60% respectively), while baking resulted in the highest fat content for faba and red kidney (1.12% and 1.39% respectively). The protein content for Phaseolus beans ranged from 21.55% in baked pinto bean to 24.92% for cooked red kidney, similar to previous work [4,35]. Faba bean protein content was approximately 30%, higher than that of the Phaseolus beans but within the anticipated range [7,8]. Extruded flours had the highest protein content for black (24.24%), faba (31.57%), and navy (24.41%), with cooking resulting in the highest protein content for pinto bean (22.47%) and red kidney bean (24.92%). The lowest protein content was found in baked samples for all Phaseolus beans, with cooking resulting in the lowest protein content for faba beans (29.73%).

### 3.2. Amino Acid Score and True Protein Digestibility

The amino acid composition for all ingredients is presented in Table 2, with the resulting amino acid scores presented in Table 3. The first limiting amino acid score for almost all processed bean samples was the sulfur amino acids, methionine and cysteine, ranging from 0.66 in extruded faba to 0.92 in cooked pinto. The one exception was cooked faba beans where tryptophan was the first limiting amino acid with a score of 0.61. It has been well documented that the limiting amino acids in beans, both raw and processed, are either the sulfur amino acids or tryptophan [5,6,7,8,36]. Interestingly, the process which resulted in the highest amino acid score differed depending on bean variety. Baking was optimal for black (0.91), faba (0.75) and navy (0.78) while cooking was best for faba (0.92) and extrusion for red kidney (0.78). Conversely, extrusion resulted in the lowest amino acid score for faba (0.66), navy (0.70) and pinto (0.80), with cooked black bean (0.83) and baked red kidney (0.72) having the lowest amino acid scores compared to other processing methods. Another study investigating the effect of cooking on the protein quality of beans found that navy and pinto beans were first limiting in tryptophan, rather than sulfur amino acids, and had lower amino acid scores for red kidney (0.70), black (0.76) and pinto (0.77), but a higher amino acid score for navy (0.83) when compared with the findings of this study [36]. A similar study determining the impact of extrusion and baking on pinto flours found similar results to the current study in that the first limiting amino acid was the sulfur amino acids, however the amino acid scores were lower than reported here (0.73 for extruded pinto vs. 0.8, and 0.7 for baked vs. 0.83) [28]. A study investigating amino acid composition of different storage proteins in Phaseolus demonstrated that modification of the ratio of these proteins altered amino acid composition [37]. It is possible that thermal processing (baking/cooking/extrusion) interacted with these protein fractions in a unique fashion, resulting in different amino acid compositions in the final product. These differences between limiting amino acid and determined amino acid scores could also be due to the use of different varieties of beans or cropping years as both of those factors can alter amino acid composition.

The in vivo and in vitro protein digestibilities are found in Table 4. A significant effect of bean type (*p* < 0.0001), processing method (*p* < 0.0001) and the interaction (*p* < 0.001) was determined for protein digestibility. For Phaseolus varieties (black, navy, pinto and red kidney) there was no significant difference in protein digestibility between extrusion and cooking (*p* > 0.05). Baking Phaseolus beans, however, resulted in significantly lower true protein digestibility compared to cooking or extrusion (*p* < 0.05). There was no significant difference in protein digestibility for processed faba bean. Extruded flours of black and pinto beans had higher digestibility than either faba or navy beans while the protein digestibility of cooked faba bean was higher than black, pinto and red kidney (*p* < 0.05). The protein digestibility of baked flours showed the greatest difference across bean varieties with faba having the highest digestibility (88.63%) and pinto had the lowest (57.58%), with significant differences between all bean varieties except navy and red kidney (69.08% and 69.12% respectively). While the protein digestibility of baked and extruded pinto flours was similar to previous work [28], all cooked Phaseolus beans had higher protein digestibility than previously reported [36]. Similar results were found in this study when comparing extruded and cooked in vivo and in vitro protein digestibilities with the in vitro digestibility being lower than that found in vivo. Baked Phaseolus flours, however, had lower digestibilities in vivo than in vitro, with a range from 4.3% lower in red kidney up to 14.14% lower in pinto. As the anti-nutritive factor concentration/activity would be identical between the in vitro and in vivo assessment of protein digestibility, this difference in baked flours is most likely due to confounding factors found in vivo, such as transit time through the intestine and digestive enzymes lacking access to the proteins, that are not present in the in vitro assay used in this study. It is also worth noting that phaseolin, the main storage protein in *Phaseolus vulgaris*, has been shown to stimulate nitrogen secretion in rats [38] and this may also act as a confounder for direct comparison between in vivo and in vitro analyses of protein digestibility.

### 3.3. In Vivo and In Vitro Protein Digestibility Corrected Amino Acid Scores

The protein digestibility corrected amino acid score (PDCAAS) is presented in Table 4. The PDCAAS ranged from a low of 47.75% for baked pinto to a high of 75.1% for cooked pinto beans. Extrusion resulted in the highest PDCAAS for black (69.74%) and red kidney (64.98%) while navy and pinto benefited the most from cooking (61.23% and 75.1% respectively). For faba, baking had the highest PDCAAS value (66.36%). Previously, cooked Phaseolus beans had PDCAAS values ranging from approximately 53% (red kidney) to 67% (navy), with lower values for red kidney, black and pinto beans than the current study [36]. In contrast baked and extruded pinto has been shown to have a higher digestibility than found in this study, 47.75% vs. 41.75% and 66.21% vs. 61.76% respectively [28,36]. The differences between these studies further highlight the potential impact of year and varietal on overall protein quality. The in vitro PDCAAS values determined in this study were consistently lower than the in vivo PDCAAS values for extruded and cooked flours, however due to the differences in protein digestibility the in vitro PDCAAS for baked Phaseolus flours were higher than those found in vivo.

Although governmental regulations require animal experimentation for the determination of protein quality, recent evidence suggests that there is a strong correlation between in vivo and in vitro measurements of protein digestibility and protein quality [28,39,40]. For that reason, this study also investigated the relationship between in vivo and in vitro protein digestibility and quality using the data generated from all samples studied. A significant correlation was found between true protein digestibility and in vitro protein digestibility (*R*^2^ = 0.5632, *p* = 0.0008). The measurements of protein quality, PDCAAS and in vitro PDCAAS, were also significantly correlated (*R*^2^ = 0.7497, *p* <0.0001). If casein was removed from the analysis, as it has a high PDCAAS and in vitro PDCAAS value, the correlation continued to be significant (*R*^2^ = 0.3762, *p* = 0.0150). Other studies using different protein sources have found similar results with correlations between PDCAAS and in vitro PDCAAS of *R*^2^ = 0.9898, 0.9280 and 0.9442 [28,39,40]. The lower *R*^2^ value found in this study, 0.3762, is due to the differences between the in vivo and in vitro results of the baked flours. When the PDCAAS/in vitro PDCAAS values for casein and the baked samples are removed, *R*^2^ = 0.8656 (*p* < 0.0001). The results of this study are in agreement with others in that in vitro analysis may be useful as an alternative to in vivo experimentation for the determination of protein quality under certain circumstances.

### 3.4. Digestible Indispensable Amino Acid Score

The digestible indispensable amino acid score (DIAAS) data is presented in Table 5. DIAAS is the newest method for determining protein quality, which was proposed as a replacement for PDCAAS in 2013 by the FAO/WHO [26]. The DIAAS values for processed beans ranged from 0.44 for baked pinto bean to 0.65 for extruded black bean. For navy bean, cooked processed method had a higher DIAAS value (0.57) and baking, like other beans, remains lower with 0.50. On the other hand, values for faba bean where different when compared to the other beans, with baking resulting in the higher DIAAS value (0.61) and extruded the lower value (0.54). As faba bean is distinct from the other beans studied (Vicia vs. Phaseolus) this may explain the difference between DIAAS values of faba and the other beans. In a study conducted by Nosworthy et al., the DIAAS values of extruded pinto flour was 0.57 and baked pinto flour was 0.39 [28]. Similar results were obtained in this study, as values for extruded and baked pinto beans were 0.61 and 0.44 respectively. Overall, PDCAAS values were higher than DIAAS values with the exception of cooked faba bean (54% vs. 59%). The calculation of DIAAS requires the use of a different reference pattern of amino acids than that of PDCAAS. An increase in the requirement of sulfur amino acids, from 25 mg/g protein in PDCAAS to 27 mg/g protein in DIAAS would lead to lower DIAAS values compared with the values obtained in PDCAAS for those protein sources limiting in sulfur amino acids [25,26]. Currently, Canada’s Food guide and the MyPlate system position cooked beans as protein sources, however in 2013 the FAO/WHO report recommended that no nutrition claim be allowed for protein sources with a DIAAS less than 0.75. In this study none of the processed beans have a DIAAS value over 0.75 and therefore not available for a source of protein content claim. A recent review of DIAAS and the potential effect of its adoption on both regulation of protein claims and consumer nutrition highlights the necessity for additional discussion of this methodology prior to widespread adoption [41].

### 3.5. Protein Efficiency Ratio

**Figure 1:**
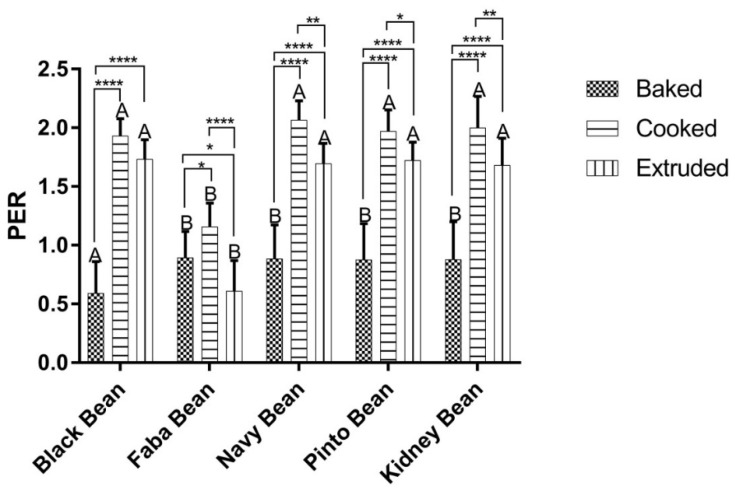
Protein Efficiency Ratio (PER) values of extruded, cooked and baked beans. Hatched bars indicate baked flour, horizontal bars are cooked flour and vertical bars are extruded flour. Mean ± SD (*n* = 10). Data were analyzed via Two-Way ANOVA with Tukey’s post-hoc test. Comparisons within pulse class are designated by lines where * *p* < 0.05, ** *p* < 0.01, **** *p* < 0.0001. Significant differences between pulses classes, but within treatment, are designated by different letters, *p* < 0.05.

Unlike PDCAAS and DIAAS, PER is a measurement of how a protein source impacts growth over a four week period and is the mandatory protein quality measurement in Canada [27]. The PER data from this study is presented in Figure 1. A significant effect of bean type (*p* < 0.0001), processing method (*p* < 0.0001) and the interaction (*p* < 0.001) was determined for PER. Processing affected the PER values of navy, pinto and kidney in the same fashion. For these three bean varieties the PER of cooked flour was significantly greater than either extruded or baked, while extruded flour was significantly higher than baked (*p* < 0.05–0.0001). A similar effect was seen in black beans, with cooking and extrusion resulting in significantly higher PER values than baking (*p* < 0.0001), however there was no difference between cooking and extrusion. While cooked faba was significantly greater than either baked (*p* < 0.05) or extruded (*p* < 0.0001), similar to the Phaseolus types, the baked faba PER was significantly higher than the extruded (*p* < 0.05), a different response than in the other beans where baked flours had the lowest PER. There was no significant differences found between extruded or cooked Phaseolus beans, however extruded and cooked faba PER was significantly lower than those of the Phaseolus beans (*p* < 0.05). In contrast, the PER of baked black bean flour was significantly lower than any other baked sample (*p* < 0.05). The PER values for the cooked Phaseolus beans are similar to those previously reported [36]. Similarly, the PER of extruded red kidney and pinto beans determined in this study are in agreement with literature values [28,42]. As a method of standardization, PER values undergo adjustment relative to the PER of casein; this normalization of PER to a casein value of 2.5 accounts for inter-laboratory and inter-run variation. These values are presented in Table 4 and indicate that for the Phaseolus beans, cooking results in the highest weight gain per unit protein consumed, followed by cooking and baking whereas for faba cooking is optimal, followed by baking and then extrusion.

## 4. Conclusions

This study demonstrated a direct effect of processing on the amino acid composition and digestibility of bean proteins, as well as overall weight gain per unit protein consumed. Although in most cases baking resulted in a better amino acid composition and higher amino acid score than either cooking or extrusion, the protein digestibility of baked bean flours was much lower than other methods potentially due to the differences in presence and/or activity of anti-nutritive factors. From this it was determined that, from a protein quality perspective, extrusion would be the most beneficial method preparing black and red kidney beans, while cooking is the most advantageous for navy and pinto beans. In many cases an outlier, being an old-world bean, the optimal method of preparing faba beans would be baking. When comparing in vivo and in vitro methods of determining protein quality, a good correlation was found between In Vitro PDCAAS and PDCAAS. These results indicate the effect of processing on bean protein quality and suggest that in vitro methods of protein digestibility analysis should be considered as a potential replacement for currently recommended in vivo rodent bioassays.

## Figures and Tables

**Table 1:** Proximate analysis of untreated, extruded, cooked and baked beans presented on a dry matter basis.

	%DM ^a^	%CF ^b^	%CP ^c^
**Casein**	93.56	0.21	92.43
**Black**			
Untreated	92.87	1.39	22.14
Extruded	96.44	0.24	24.24
Cooked	100.00	1.61	22.88
Baked	98.50	1.36	22.21
**Faba**			
Untreated	92.15	1.03	29.03
Extruded	96.79	1.04	31.57
Cooked	95.67	1.10	29.73
Baked	97.78	1.12	30.11
**Navy**			
Untreated	92.49	1.33	22.91
Extruded	96.81	1.50	24.41
Cooked	98.26	1.71	24.36
Baked	97.86	1.70	23.70
**Pinto**			
Untreated	91.68	1.31	21.34
Extruded	96.85	0.27	22.40
Cooked	95.13	1.60	22.47
Baked	95.13	1.06	21.55
**Red Kidney**			
Untreated	92.92	1.05	24.15
Extruded	96.19	0.61	24.49
Cooked	98.56	1.37	24.92
Baked	98.90	1.39	23.82

^a^ DM = dry matter content. ^b^ CF = crude fat determined by hexane extraction ^c^ CP = crude protein = nitrogen content (determined by DUMAS analysis) × 6.25.

**Table 2:** Amino acid composition of extruded, cooked and baked beans.

	ASP	THR	SER	GLU	PRO	GLY	ALA	CYS	VAL	MET	ILE	LEU	TYR	PHE	HIS	LYS	ARG	TRP
**Casein**	7.78	3.35	5.64	20.05	9.77	1.35	3.16	0.78	5.02	1.45	3.84	8.39	4.83	4.59	2.74	6.96	3.12	1.08
**Black**																		
Extruded	2.61	0.98	1.61	3.4	0.87	0.87	0.97	0.23	1.24	0.27	0.95	2.02	0.58	1.38	0.83	1.36	1.29	0.25
Cooked	3.11	1.07	1.55	3.28	1.36	0.91	1.18	0.24	1.13	0.23	0.91	1.83	0.82	1.47	0.9	1.68	1.53	0.28
Baked	2.5	1.03	1.48	3.06	0.94	0.78	1.08	0.25	1.05	0.25	0.81	1.8	0.07	1.24	0.8	1.3	1.43	0.25
**Faba**																		
Extruded	3.27	0.98	1.53	4.74	1.23	1.09	1.32	0.27	1.25	0.24	1.05	2.19	0.9	1.26	0.88	1.6	2.7	0.28
Cooked	3.04	0.91	1.47	4.47	1.04	1.07	1.29	0.3	1.16	0.21	0.99	2.19	0.8	1.12	0.83	1.77	2.74	0.19
Baked	3.18	0.91	1.49	4.59	1.24	1.09	1.34	0.32	1.26	0.24	1.03	2.13	0.78	1.18	0.82	1.82	2.97	0.25
**Navy**																		
Extruded	2.91	0.99	1.5	3.66	0.7	0.87	1.12	0.2	1.25	0.21	0.99	1.94	0.64	1.25	0.84	1.43	1.43	0.27
Cooked	3.04	1.05	1.65	3.45	0.74	0.91	1.17	0.2	1.26	0.22	1.05	2.12	0.68	1.42	0.87	1.67	1.44	0.3
Baked	2.65	0.94	1.44	3.25	0.73	0.82	1.05	0.21	1.16	0.24	0.91	1.86	0.6	1.11	0.79	1.28	1.32	0.28
**Pinto**																		
Extruded	2.81	0.83	1.37	3.5	0.74	0.75	1.02	0.19	1.04	0.25	0.86	1.97	0.62	1.34	0.82	1.3	1.19	0.25
Cooked	2.73	0.97	1.57	3.17	0.71	0.82	0.98	0.22	1.06	0.27	1.03	2.02	0.7	1.38	0.83	1.62	1.37	0.25
Baked	2.53	0.85	1.31	3.22	0.6	0.78	1.04	0.19	1.06	0.24	0.82	1.75	0.65	1.09	0.76	1.34	1.37	0.28
**RedKidney**																		
Extruded	3	0.99	1.55	3.91	0.65	0.88	1.15	0.22	1.19	0.24	0.96	2	0.66	1.31	0.9	1.46	1.43	0.26
Cooked	3.16	1.07	1.71	3.9	0.75	0.93	1.21	0.24	1.27	0.24	1.03	2.21	0.72	1.43	0.96	1.8	1.49	0.3
Baked	3.05	1.02	1.62	3.89	0.78	0.94	1.25	0.23	1.22	0.2	1.04	2.1	0.68	1.36	0.91	1.45	1.47	0.3

**Table 3:** Amino acid score of extruded, cooked and baked beans.

	THR	VAL	MET + CYS	ILE	LEU	PHE + TYR	HIS	LYS	TRP
**Casein**	1.14	1.66	**1.03**	1.59	1.47	1.73	1.67	1.39	1.13
**Black**									
Extruded	1.23	1.51	**0.85**	1.45	1.31	1.33	1.87	1.01	0.98
Cooked	1.38	1.42	**0.83**	1.42	1.21	1.59	2.07	1.26	1.10
Baked	1.39	1.37	**0.91**	1.31	1.25	0.95	1.92	1.02	1.03
**Faba**									
Extruded	0.95	1.17	**0.66**	1.23	1.08	1.12	1.51	0.90	0.84
Cooked	0.94	1.16	**0.72**	1.24	1.17	1.07	1.54	1.07	**0.61**
Baked	0.91	1.22	**0.75**	1.25	1.10	1.06	1.47	1.06	0.78
**Navy**									
Extruded	1.23	1.51	**0.70**	1.49	1.24	1.27	1.88	1.05	1.05
Cooked	1.30	1.51	**0.71**	1.56	1.34	1.40	1.91	1.20	1.14
Baked	1.19	1.43	**0.78**	1.41	1.22	1.17	1.80	0.95	1.09
**Pinto**									
Extruded	1.12	1.37	**0.80**	1.42	1.37	1.44	1.99	1.03	1.06
Cooked	1.33	1.42	**0.92**	1.71	1.43	1.54	2.05	1.30	1.05
Baked	1.22	1.47	**0.83**	1.42	1.29	1.35	1.96	1.13	1.26
**RedKidney**									
Extruded	1.24	1.44	**0.78**	1.46	1.28	1.33	2.01	1.07	1.02
Cooked	1.28	1.48	**0.77**	1.50	1.36	1.39	2.05	1.26	1.10
Baked	1.28	1.47	**0.72**	1.57	1.35	1.37	2.03	1.06	1.14

Bolded values indicate the first limiting amino acid. The reference pattern used to calculate the amino acid scores was as followed (mg/g protein): Thr—34, Val—35, Met + Cys—25, Ile—28, Leu—66, Phe + Tyr—63, His—19, Lys—58, Trp—11.

**Table 4:** Adjusted protein efficiency ratio, protein digestibility-corrected amino acid scores and in vitro protein digestibility-corrected amino acid Scores of extruded, cooked and baked beans.

	AAS ^a^	TPD ^b^	IVPD ^c^	PDCAAS ^d^	IVPDCAAS ^e^	Adj. PER ^f^
**Casein**	1.03	97.31 (0.61)	90.73 (2.52)	100	93.54	2.5
**Black**						
Extruded	0.85	82.01 (2.27) ^aA^	79.42 (0.54)	69.74	67.55	1.27
Cooked	0.83	81.66 (2.12) ^aAC^	75.34 (1.00)	67.54	62.31	1.42
Baked	0.91	63.55 (4.11) ^bA^	74.26 (0.09)	57.52	67.22	0.43
**Faba**						
Extruded	0.66	87.60 (3.77) ^aB^	82.22 (0.63)	58.01	54.45	0.45
Cooked	0.61	88.49 (3.99) ^aB^	81.41 (0.00)	54.14	49.81	0.85
Baked	0.75	88.63 (4.29) ^aB^	76.79 (0.45)	66.36	57.49	0.66
**Navy**						
Extruded	0.7	87.41 (2.56) ^aB^	79.51 (0.09)	60.82	55.33	1.24
Cooked	0.71	86.02 (2.73) ^aAB^	77.06 (2.53)	61.23	54.86	1.52
Baked	0.78	69.08 (4.28) ^bC^	78.51 (0.36)	53.62	60.95	0.65
**Pinto**						
Extruded	0.8	82.53 (2.08) ^aA^	80.95 (1.0)	66.21	64.95	1.26
Cooked	0.92	82.07 (4.51) ^aAC^	76.70 (0.54)	75.1	70.19	1.45
Baked	0.83	57.58 (3.71) ^bD^	71.72 (0.27)	47.75	59.48	0.64
**RedKidney**						
Extruded	0.78	83.21 (1.91) ^aAB^	81.95 (0.00)	64.98	63.99	1.23
Cooked	0.77	80.67 (2.09) ^aC^	82.40 (0.45)	62.4	63.74	1.47
Baked	0.72	69.12 (4.84) ^bC^	73.44 (0.00)	50.1	53.24	0.65

Numbers in parentheses indicate SD where applicable. TPD was analyzed via Two-Way ANOVA with Tukey’s post-hoc test. Means followed by different letters (small in the same pulse class and large in the same treatment) indicate a significant difference between samples (*p* < 0.05). ^a^ AAS = amino acid score ^b^ %TPD = % true protein digestibility ^c^ IVPD = in vitro protein digestibility ^d^ PDCAAS = protein digestibility corrected amino acid score ^e^ In Vitro PDCAAS = in vitro protein digestibility corrected amino acid score ^f^ Adj. PER = Adjusted PER. *n* = 10 for Adj. PER and %TPD; *n* = 2 for IVPD and *n* = 1 for AAS, PDCAAS, In Vitro PDCAAS. PDCAAS is calculated as the product of AAS and %TPD while In Vitro PDCAAS is the product of AAS and IVPD.

**Table 5:** Digestible indispensable amino acid scores of extruded, cooked and baked beans.

	THR	VAL	MET + CYS	ILE	LEU	PHE + TYR	HIS	LYS	TRP	DIAAS ^a^
**Casein**	1.22	1.31	**0.93**	1.35	1.43	2.04	1.54	1.37	1.42	0.93
**Black**										
Extruded	1.11	1.01	**0.65**	1.04	1.07	1.32	1.46	0.84	1.04	0.65
Cooked	1.24	0.94	**0.63**	1.01	0.99	1.57	1.60	1.05	1.16	0.63
Baked	0.97	0.71	**0.53**	0.73	0.79	0.73	1.16	0.66	0.85	0.53
**Faba**										
Extruded	0.91	0.83	**0.54**	0.94	0.95	1.19	1.26	0.80	0.95	0.54
Cooked	0.91	0.84	**0.59**	0.96	1.03	1.15	1.29	0.97	0.70	0.59
Baked	0.88	0.88	**0.61**	0.97	0.97	1.13	1.23	0.96	0.90	0.61
**Navy**										
Extruded	1.18	1.07	**0.56**	1.14	1.09	1.35	1.56	0.93	1.19	0.56
Cooked	1.22	1.05	**0.57**	1.18	1.16	1.45	1.56	1.05	1.27	0.57
Baked	0.90	0.81	**0.50**	0.85	0.84	0.98	1.18	0.67	0.98	0.50
**Pinto**										
Extruded	1.02	0.92	**0.61**	1.02	1.13	1.44	1.56	0.86	1.14	0.61
Cooked	1.20	0.95	**0.70**	1.23	1.17	1.54	1.60	1.09	1.12	0.70
Baked	0.77	0.69	**0.44**	0.72	0.74	0.94	1.07	0.66	0.94	0.44
**RedKidney**										
Extruded	1.13	0.97	**0.60**	1.06	1.07	1.34	1.59	0.91	1.10	0.60
Cooked	1.13	0.97	**0.58**	1.06	1.10	1.36	1.57	1.04	1.15	0.58
Baked	0.97	0.83	**0.46**	0.95	0.93	1.15	1.33	0.75	1.02	0.46

^a^ DIAAS = Digestible Indispensable Amino Acid Score. DIAAS was calculated using true protein digestibility. Bolded values reflect first limiting amino acid.
